# Beyond entertainment: cultural–psychological mechanisms underlying the reduction of state anxiety in heritage-based serious games

**DOI:** 10.3389/fpsyg.2026.1854835

**Published:** 2026-08-17

**Authors:** Bin Wang, JiaXin Zou, JianFeng Wang

**Affiliations:** 1 College of Design, Graduate School, Hanyang University, Seoul, Republic of Korea; 2 Department of Environmental Art Design, Zibo Polytechnic University, Zibo, China

**Keywords:** cultural identity, flow, heritage-based serious games, state anxiety, virtual heritage place attachment

## Abstract

Anxiety has become an increasingly prominent mental health concern among university students, highlighting the need for effective and engaging intervention approaches. Although serious games have been shown to reduce anxiety, existing research has primarily attributed these effects to general processes such as distraction, relaxation, and immersion, leaving the underlying psychological mechanisms insufficiently specified—particularly in culturally embedded contexts. Drawing on the Stimulus–Organism–Response (SOR) framework and Self-Determination Theory (SDT), this study examines whether heritage-based serious games reduce State Anxiety through a culturally grounded psychological pathway. It proposes that Heritage Narrative Involvement, Perceived Authenticity, and Perceived Autonomy influence emotional outcomes through Cultural Identity, Virtual Heritage Place Attachment, and Flow. A three-study design was employed. Study 1 used a randomized active-controlled experiment to identify the short-term effect of cultural embedding on State Anxiety. Study 2 tested the proposed mechanism using large-sample data, combining structural modeling and predictive analysis to enhance robustness. Study 3 used qualitative interviews to further explain how these mechanisms are experienced by players. Results indicate that heritage-based serious games produce stronger reductions in State Anxiety than general gameplay. Importantly, these effects are not solely attributable to distraction or engagement, but emerge through a structured psychological process involving cultural internalization, place-based attachment, and immersive experience. These findings contribute to research on emotion regulation and digital interventions by demonstrating that cultural content can function as an active psychological resource, rather than merely contextual information, in shaping emotional outcomes.

## 1 Introduction

Anxiety has become one of the most prevalent mental health concerns worldwide, exerting particularly significant effects on young adults undergoing academic, social, and career transitions (Nochaiwong et al., 2021). For university students approaching graduation, multiple stressors—including academic competition, employment uncertainty, financial pressure, and family expectations—often intersect and accumulate, further intensifying immediate emotional experiences such as tension, worry, and unease (Smail-Crevier et al., 2025; Korkut and Altıntas¸, 2024). Within this context, State Anxiety warrants particular attention, as it reflects a situational and transient negative emotional state that can directly impair attention, decision-making, subjective well-being, and adaptive functioning in daily life (White et al., 2018). As the demand for low-burden, accessible forms of emotional support suitable for everyday, non-clinical settings continues to grow, identifying digital interventions that may help alleviate State Anxiety has become an increasingly important issue in digital mental health research.

Against this backdrop, Serious Games have been increasingly explored as potentially promising digital interventions for anxiety-related outcomes (D’Alfonso, 2020). By integrating emotion regulation, behavioral engagement, and cognitive activation within interactive and immersive environments, they may support engagement and sustained participation (Lau et al., 2017). Their effects, however, are not uniform across game types. Exergames have been reported to produce effects comparable to physical exercise, whereas CBT-based games and exergames have been found to outperform no-intervention conditions (Abd-Alrazaq et al., 2022). Games incorporating biofeedback or neurofeedback may be particularly promising because they convert physiological signals into real-time adaptive feedback (Gómez-León, 2025). Emotion regulation–oriented Serious Games have also shown potential effectiveness across general, at-risk, and clinical populations (Schoneveld et al., 2018; Schoneveld et al., 2020). Yet the mechanisms underlying these effects remain underspecified. Existing research mainly invokes generic processes such as distraction, relaxation, behavioral activation, or immersion, offering limited leverage for explaining whether and how different game contents and experiential designs produce qualitatively distinct psychological effects. Thus, the literature has progressed further in establishing that Serious Games can work than in explaining why specific types work through distinct mechanisms.

At the same time, research on Digital Heritage and heritage-based games shows that culturally grounded digital experiences may strengthen engagement, perceived meaning, immersion, and continuance intention (Li et al., 2024; Lin et al., 2025). In Intangible Cultural Heritage (ICH) settings, digital games can further enhance the accessibility and attractiveness of cultural content while fostering Cultural Identity, hedonic experience, and sustained engagement (Qiu et al., 2024; Wang and Men, 2025). Yet this literature has predominantly treated heritage-based games as instruments of cultural dissemination, educational display, or continuance intention. Far less attention has been paid to whether such experiences may also operate as psychological resources. As a result, while existing work has made substantial progress in explaining cultural dissemination outcomes, it remains underdeveloped in accounting for short-term emotional outcomes, especially the mechanisms through which State Anxiety may be reduced.

This gap has potentially important theoretical implications. If anxiety reduction in games is attributable only to relaxation or attentional distraction, then the contribution of heritage-based Serious Games cannot be reduced to showing that they are effective as well. Their theoretical value may lies in clarifying whether culturally embedded game experiences operate through mechanisms that extend beyond general gaming effects. Heritage-based Serious Games may be particularly suitable for this purpose because they combine heritage narratives, authenticity cues, spatial construction, and autonomous participation within a unified experiential system. In such contexts, players may not merely encounter cultural symbols as external information; but may also actively construct, organize, manage, and repeatedly inhabit culturally meaningful virtual spaces. Cultural content may therefore function not only as representational material, but as a psychological resource internalized through experience. Thus, the central issue is not simply whether heritage-based Serious Games reduce State Anxiety, but whether their emotional effects are partly produced through a culturally specific cultural–psychological mechanism.

Building on this premise, the present study reconceptualizes heritage-based Serious Games as culturally embedded digital experiential environments rather than simply as vehicles for cultural dissemination or entertainment products with traditional elements. This shift allows the analytical focus to move whether users encounter cultural content to how cultural experience is psychologically transformed. Within such environments, players are exposed not only to heritage narratives, cultural aesthetics, and authenticity cues, but also to autonomous spatial construction and sustained interaction, through which cultural content may become self-relevant and personally meaningful. The emotional effects of heritage-based Serious Games, therefore, may not be fully explained by pleasure, slow pacing, or generic immersion. Rather, they are more likely to arise through interrelated psychological states such as Cultural Identity, Virtual Heritage Place Attachment, and Flow. Heritage-based Serious Games may thus function not only as media for cultural dissemination, but also as structured experiential contexts with emotion-regulatory potential.

Building on the above perspective, this study focuses on university students approaching graduation and examines whether heritage-based Serious Games may alleviate their State Anxiety, as well as the mechanisms through which this effect is achieved. Specifically, this study addresses the following research questions:

RQ1: To what extent do heritage-based Serious Games reduce university students’ State Anxiety in the short term?

RQ2: Is this anxiety-reduction effect mediated by internal psychological mechanisms activated by culturally embedded game experiences?

RQ3: Do these internal psychological mechanisms form an ordered chain that jointly helps explain how heritage-based Serious Games influence State Anxiety?

To address these questions, this study adopts a three-study evidence-chain design. Study 1 uses a randomized controlled experiment with an active control group to help distinguish the cultural embedding effect from general Serious Game effects and to test the short-term effect of heritage-based Serious Games on State Anxiety. Study 2 uses a large sample to examine the proposed psychological mechanism and strengthen the robustness of the findings through complementary analyses. Study 3 uses semi-structured interviews to clarify how players experience authenticity, develop Cultural Identity, form Virtual Heritage Place Attachment, and enter immersive states during gameplay. By integrating effect identification, mechanism validation, and experiential interpretation, the study provides a more integrated and nuanced account of how heritage-based Serious Games may serve as culturally grounded emotional interventions.

This study seeks to make three contributions. First, it reconceptualizes heritage-based Serious Games as culturally embedded experiential contexts with psychological consequences, rather than merely as vehicles for cultural dissemination or continuance intention. Second, it shifts the focus from whether such games are effective to how they may contribute to reductions in State Anxiety by identifying the roles of Cultural Identity, Virtual Heritage Place Attachment, and Flow in the underlying psychological process. Third, it develops and empirically examines a theoretical proposition: that the emotional benefits of heritage-based Serious Games may arise partly not only from general gameplay-related relaxation, but also from a culturally specific psychological pathway shaped by Heritage Narrative Involvement, Perceived Authenticity, and autonomy-supportive spatial construction. The study therefore contributes to research on digital interventions, emotion regulation, and culturally grounded psychological processes by suggesting that cultural content can function as an active psychological resource.

## 2 Literature review

### 2.1 Theoretical foundations

#### 2.1.1 From general game effects to culturally embedded experiential effects

Existing research suggests that Serious Games reduce anxiety through distraction, relaxation, behavioral activation, and immersion, and reviews further support their overall effectiveness. Yet this literature mainly explains why games work as games, emphasizing general features such as entertainment, feedback, and interactivity while paying less attention to the differentiated psychological consequences of content type and experiential design (Abd-Alrazaq et al., 2022; Wols et al., 2024). This limitation is particularly salient for culturally embedded Serious Games, especially heritage-based ones, whose effects may exceed general stress-reduction logic. At the same time, Digital Heritage research shows that heritage-based digital experiences shape not only knowledge acquisition, but also affective experience, immersion, continuance intention, and culture-related psychological constructs. In ICH games and immersive cultural experiences, affective design, cultural perception, and place-based experience have been increasingly identified as key determinants of user response (Lin et al., 2025; Bai et al., 2025; Qiu et al., 2024). Together, these findings suggest that the emotional effects of heritage-based Serious Games may derive not only from generic mechanisms such as relaxation or distraction, but also from culturally grounded psychological processes shaped by heritage narratives, authenticity cues, spatial management, and autonomous participation. Explaining their effects on State Anxiety therefore requires an integrative framework that captures both the psychological processing of cultural stimuli and the internalization of cultural content through autonomous participation, for which the “perception–place–behavior” chain in recent Digital Heritage research provides direct theoretical inspiration (Deng et al., 2026).

#### 2.1.2 SOR as an overarching framework: cultural stimuli, internal states, and emotional outcomes

To explain how heritage-based Serious Games shape players’ immediate emotional states, this study adopts the Stimulus–Organism–Response (SOR) framework as its overarching analytical model (Arora, 1982). SOR is valuable because it links external experiential features, internal psychological states, and emotional outcomes within a unified process, avoiding a reductive stimulus–outcome account. Recent research on Intangible Cultural Heritage (ICH) games suggests that this framework is particularly suitable for explaining the relationships among cultural content, interaction characteristics, and user psychological responses. For instance, Bai et al. (2025) and Qiu et al. (2024) applied SOR in ICH-based Serious Games and ICH-based VR games, respectively, demonstrating its relevance for culturally oriented game contexts. In the present study, the stimulus layer consists of Heritage Narrative Involvement, Perceived Authenticity, and Perceived Autonomy; the organism layer comprises Cultural Identity, Virtual Heritage Place Attachment, and Flow; and the response layer is State Anxiety. This mapping reflects a broader assumption in Digital Heritage research that digital cultural experiences first shape perception, affect, and identity, and only then produce behavioral or emotional outcomes. Supporting this view, Deng et al. (2026) proposed a “perception–place–behavior” model of digital heritage experience, and Sun et al. (2026) showed that Cultural Identity and sense of place mediate the relationship between external experience and later outcomes. However, although SOR captures how cultural stimuli influence outcomes through internal states, it is less precise in explaining why autonomous construction and self-directed participation are especially likely to foster cultural internalization and place-based attachment. SOR therefore provides a necessary macro-level framework, but is not sufficient on its own to explain how participation becomes internalized identification.

#### 2.1.3 SDT as a critical complement: autonomy-supportive experiences and cultural internalization

To address these limitations, this study introduces Self-Determination Theory (SDT) as a critical complementary mechanism. SDT holds that autonomy is a basic psychological need; when individuals experience choice, agency, and volition, they are more likely to internalize external content as self-endorsed values and to engage more deeply (Ryan and Deci, 2024). Consistent with this view, research on digital game-based learning shows that satisfaction of basic psychological needs, especially autonomy, is positively associated with higher Flow (Lüking et al., 2023). Studies on Serious Game design likewise indicate that satisfying autonomy, competence, and relatedness strengthens engagement and motivational quality (Yang et al., 2025). In heritage-based Serious Games, autonomy is not merely operational freedom, but the capacity to construct courtyards, arrange cultural spaces, and manage virtual heritage environments in a self-directed way. Under these conditions, cultural content is not simply received as information, but becomes internalized as self-relevant experience through construction, choice, and expression. SDT is therefore introduced not as a parallel framework to SOR, but to clarify what SOR alone cannot fully explain: why autonomy-supportive experiences are especially conducive to Cultural Identity, Virtual Heritage Place Attachment, and high-quality immersive engagement.

### 2.2 Conceptual definitions of core variables

Heritage Narrative Involvement refers to the psychological state in which players are continuously drawn into ICH-related story contexts, cultural cues, and narrative structures during gameplay (Yu et al., 2025). Rather than merely receiving cultural information, players enter specific historical–cultural contexts through narrative progression, generating cognitive attention, emotional engagement, and meaning-making. In the present study, it captures the extent to which players immerse themselves in the ICH cultural world and marks a key point at which cultural content shifts from external presentation to internalized experience (Lin et al., 2025).

Perceived Authenticity refers to players’ subjective judgments that the ICH content, traditional spaces, cultural symbols, and related experiences presented in the game are credible, culturally grounded, and historically legitimate. Consistent with recent heritage research, authenticity here does not denote mere objective “realness,” but the perceived historical depth, cultural grounding, and sense of presence of the experience (Ries and Schwan, 2024). It therefore extends beyond visual realism or surface fidelity to whether the cultural content is experienced as reliable, coherent, and culturally meaningful. In this study, Perceived Authenticity reflects players’ overall evaluation of the cultural quality and credibility of heritage-based Serious Games and serves as a key antecedent of Cultural Identity and place-based emotional attachment (Yi et al., 2024).

Perceived Autonomy refers to the degree of choice, self-direction, and volitional control players experience during gameplay, as reflected in their ability to construct, arrange, manage, and interact according to personal preferences rather than under full external constraint (Yang et al., 2025). In the present study, it denotes not merely operational freedom, but the psychological experience of self-directed construction of virtual heritage spaces. This conceptualization is aligned with recent SDT-based game research, which treats autonomy as a core condition for self-expression, intrinsic motivation, and high-quality engagement (Tyack and Mekler, 2024). Perceived Autonomy thus captures the extent of active participation in the cultural experience process and constitutes a key condition for both cultural internalization and high-quality immersive engagement (Lüking et al., 2023).

Cultural Identity refers to the extent to which players develop identification, acceptance, and psychological connection with the values, aesthetic meanings, historical memories, and symbolic significance embedded in ICH during gameplay (Chan et al., 2024; Sun et al., 2026). It reflects whether cultural content is experienced as self-relevant, endorsable, and psychologically acceptable. In this study, Cultural Identity is not simply cognitive understanding of cultural knowledge, but the internalization of cultural content as subjectively meaningful psychological outcome. It therefore serves as a key mediating variable linking cultural stimuli to subsequent place-based emotions and experiential states (Chan et al., 2024).

Virtual Heritage Place Attachment is an environmental psychology concept describing the emotional and cognitive bonds individuals form with places (Zhu et al., 2025). Virtual Heritage Place Attachment refers to the emotional attachment, sense of belonging, and perceived place meaning that players develop toward ICH-based virtual spaces through sustained gameplay (Chan et al., 2024). It marks a shift in which the game environment is experienced not merely as a functional backdrop, but as a culturally valued and emotionally meaningful place of subjective belonging (Qiu et al., 2025). In the present study, it captures the transformation of ICH-related spaces from viewed environments into emotionally connected places and thus reflects the spatialization and emotionalization of cultural content.

Flow refers to an optimal experiential state marked by intense concentration, deep immersion, intrinsic reward, and reduced self-awareness during gameplay (Oliveira and Hamari, 2025; Lu et al., 2025). Because attention is strongly focused on the ongoing activity, external distraction and negative rumination are reduced. In this study, Flow represents a high-quality experiential state in heritage-based Serious Games and functions as a proximal mechanism linking cultural experiential factors to emotional outcomes (Lüking et al., 2023).

State Anxiety refers to a transient state of tension, unease, worry, and heightened arousal arising in specific situations. Unlike trait anxiety, it is situational, momentary, and fluctuating, and thus more directly influenced by immediate environments and ongoing experience. In this study, State Anxiety is the final outcome variable, used to assess whether heritage-based Serious Games alleviate immediate negative emotional states and thereby demonstrate potential for real-time emotion regulation (Sánchez-Vincitore et al., 2025; Gómez-León, 2025).

### 2.3 Research hypotheses

#### 2.3.1 Cultural internalization mechanism

In heritage-based Serious Games, Cultural Identity depends less on mere exposure to cultural content than on whether such content is experienced as accessible, credible, and self-relevant. Heritage Narrative Involvement converts fragmented cultural information into coherent and meaningful experience, thereby deepening cognitive and emotional engagement. Prior research shows that narrative contexts facilitate self-consistency and identity connection with Intangible Cultural Heritage (ICH) subjects (Guo et al., 2022), while Digital Heritage studies suggest that gamified narratives immerse users in heritage content and strengthen cultural experience (Rizvic et al., 2024). Perceived Authenticity provides the legitimacy basis for accepting such content, extending beyond visual realism to whether cultural representation is grounded in credible cultural foundations and historical legitimacy. Empirical evidence indicates that Perceived Authenticity promotes localized identity, cultural self-enhancement, and heritage responsibility (Yi et al., 2024), while strengthening emotional and cognitive responses to historical places (Jiang et al., 2026). Perceived Autonomy, in turn, supports cultural internalization by transforming externally imposed task demands into self-relevant experience. From an SDT perspective, autonomy reflects volitional participation rather than mere operational freedom and thus facilitates internalization and high-quality engagement. Consistent with this logic, autonomy satisfaction is associated with more positive engagement experiences (Lüking et al., 2023), and autonomy-supportive design enhances active participation in and internalization of learning and cultural content (Yang et al., 2025). When players can arrange spaces, determine management strategies, and construct virtual cultural environments according to their own preferences, cultural content is more likely to become self-relevant, thereby strengthening Cultural Identity (Tyack and Mekler, 2024). Based on the above analysis, this study proposes:

*H1*: Heritage Narrative Involvement has a positive effect on Cultural Identity.

*H2*: Perceived Authenticity has a positive effect on Cultural Identity.

*H3*: Perceived Autonomy has a positive effect on Cultural Identity.

#### 2.3.2 Placemaking mechanism of virtual heritage spaces

Virtual Heritage Place Attachment depends not on spatial representation alone, but on whether virtual space is experienced as culturally credible, psychologically meaningful, and open to autonomous participation. Perceived Authenticity provides the cultural depth and legitimacy required for virtual space to become a meaningful place. In heritage-based Serious Games, authenticity extends beyond visual realism to whether space is grounded in credible cultural foundations and historical legitimacy. Prior research has shown a stable positive association between authenticity and place attachment (Qiu et al., 2025), and heritage environments perceived as authentic and culturally grounded are more likely to attract emotional investment (Zhu et al., 2025). Cultural Identity provides the psychological content through which such attachment develops: whereas it reflects endorsement of cultural values and symbolic meanings, place attachment reflects emotional belonging to the spaces in which those meanings are embodied. In the present study, the proposed direction from Cultural Identity to Virtual Heritage Place Attachment is grounded in a cultural internalization–placemaking logic. Cultural Identity is conceptualized as a broader psychological recognition and internalization of cultural meanings, whereas Virtual Heritage Place Attachment refers to a more space-specific emotional bond with the virtual heritage environment. From this perspective, players are unlikely to form attachment to a virtual heritage space merely because it is visually or spatially represented. Rather, such attachment is more likely to emerge when the space is first interpreted as culturally meaningful, credible, and personally relevant. Cultural Identity therefore provides the meaning basis through which virtual space can be transformed from a represented environment into an emotionally meaningful place. This relationship has been observed across heritage experiences (Chan et al., 2024), with both constructs often operating jointly as mediators of subsequent responses (Deng et al., 2026). Perceived Autonomy further enables players to appropriate virtual space as personally meaningful place through active construction. SDT-based research suggests that autonomy satisfaction strengthens intrinsic engagement and subjective connection in digital environments (Tyack and Mekler, 2024), while heritage studies indicate that place identity and place attachment depend not only on representation, but also on participation, control, and self-expression within space (Shi and Tan, 2025). Thus, in heritage-based Serious Games, Perceived Authenticity establishes virtual environments as culturally meaningful places, Cultural Identity endows them with emotional significance, and autonomous participation transforms them into “my place,” thereby facilitating Virtual Heritage Place Attachment. Although the reverse relationship may also occur during repeated or long-term interaction, where attachment to a virtual heritage space may further reinforce Cultural Identity, the present model focuses on the short-term experiential process through which culturally embedded gameplay is interpreted, internalized, and spatialized. Accordingly, Cultural Identity is proposed as an antecedent of Virtual Heritage Place Attachment in this study. Based on the above analysis, this study proposes:

*H4*: Perceived Authenticity positively predicts Virtual Heritage Place Attachment.

*H5*: Cultural Identity positively predicts Virtual Heritage Place Attachment.

*H6*: Perceived Autonomy positively predicts Virtual Heritage Place Attachment.

#### 2.3.3 Immersion-based restorative mechanism

In heritage-based serious games, emotional restoration may be supported through the interplay among autonomy support, place attachment, and high-quality immersion. First, flow is more likely to occur in contexts characterized by freedom of action, self-directed pacing, and internally coherent participation. Prior research has shown that autonomy satisfaction is an important predictor of flow experience in digital gamified environments (Lüking et al., 2023), while autonomy-supportive design, perceived control over participation, and high-quality engagement also contribute to the formation of flow (Oliveira and Hamari, 2025). Meanwhile, deep immersion depends not only on task design but also on the sustained emotional connection between individuals and the environment (Oliveira and Hamari, 2025). Heritage research has also indicated that authenticity and place attachment jointly constitute an important basis for deeper experiential responses (Zhu et al., 2025). Therefore, higher levels of Perceived Autonomy and Virtual Heritage Place Attachment are expected to may facilitate the formation of Flow. Second, Virtual Heritage Place Attachment and Flow jointly constitute proximal mechanisms for alleviating State Anxiety. Research on digital environments suggests that when individuals develop emotional connections with a space, they are more likely to experience stability, familiarity, and psychological settlement, thereby demonstrating better well-being and lower psychological distress (Lassota et al., 2025). Environmental psychology research also indicates that Virtual Heritage Place Attachment can enhance well-being by satisfying psychological needs and strengthening emotional stability (Yang and Li, 2025). At the same time, research on serious games and anxiety suggests that high-quality immersion is an important experiential mechanism through which digital games alleviate anxiety (Abd-Alrazaq et al., 2022), while high-flow conditions are associated with stronger focused engagement and reduced distracting processing (Joessel et al., 2024). Therefore, both Virtual Heritage Place Attachment and Flow are expected to contribute to lower State Anxiety. Based on this reasoning, this study proposes:

*H7*: Perceived Autonomy has a positive effect on Flow.

*H8*: Virtual Heritage Place Attachment has a negative effect on State Anxiety.

*H9*: Virtual Heritage Place Attachment has a positive effect on Flow.

*H10*: Flow has a negative effect on State Anxiety.

### 2.4 Model development

**Figure 1:**
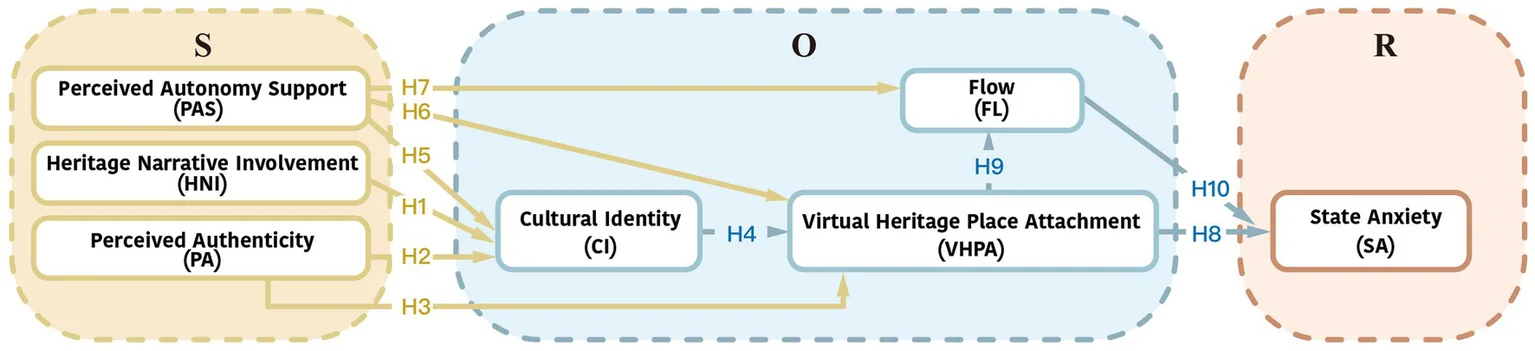
Research model diagram.

This study proposes a conceptual model that adopts the Stimulus–Organism–Response (SOR) framework as its overarching structure and integrates Self-Determination Theory (SDT) as a complementary mechanism to explain how heritage-based Serious Games influence State Anxiety through internal psychological transformation. As shown in Figure 1, Heritage Narrative Involvement, Perceived Authenticity, and Perceived Autonomy form the stimulus layer; Cultural Identity, Virtual Heritage Place Attachment, and Flow constitute the organism layer; and State Anxiety is the response layer. The central claim is that the emotional benefits of heritage-based Serious Games derive not merely from generic relaxation effects of gameplay, but from the transformation of cultural content into Cultural Identity, place-based attachment, and immersive engagement through narrative involvement, authenticity cues, and autonomous participation. In this model, SOR provides the macro-level structural pathway, whereas SDT clarifies how autonomous participation facilitates cultural internalization and high-quality experiential engagement.

## 3 Methodology

### 3.1 Research design

This study adopts a three-study, evidence-chain design (see Supplementary Figure 1). Study 1 employs an AB-group randomized controlled experiment to test the short-term effect of heritage-based Serious Games on university students’ State Anxiety under small-sample, high-control conditions. Manipulation checks are used to verify activation of the key stimulus variables. Group A represents the heritage-based Serious Game condition, and Group B a non-heritage Serious Game active control. The two conditions are matched on gameplay duration, core management mechanics, operational load, and interaction process, while differing systematically in cultural embedding, thereby isolating the ICH cultural embedding effect from the general Serious Game effect. Study 2 uses large-sample real-user data and an integrated SEM–ANN approach to test structural relationships, assess robustness, and evaluate predictive performance. Study 3 draws on semi-structured interviews to clarify how key mechanism variables emerge in actual gameplay and how participants subjectively relate these experiences to reductions in State Anxiety, thus enhancing the explanatory depth and contextual validity of the model.

### 3.2 Experimental context and stimulus materials

**Figure 2:**
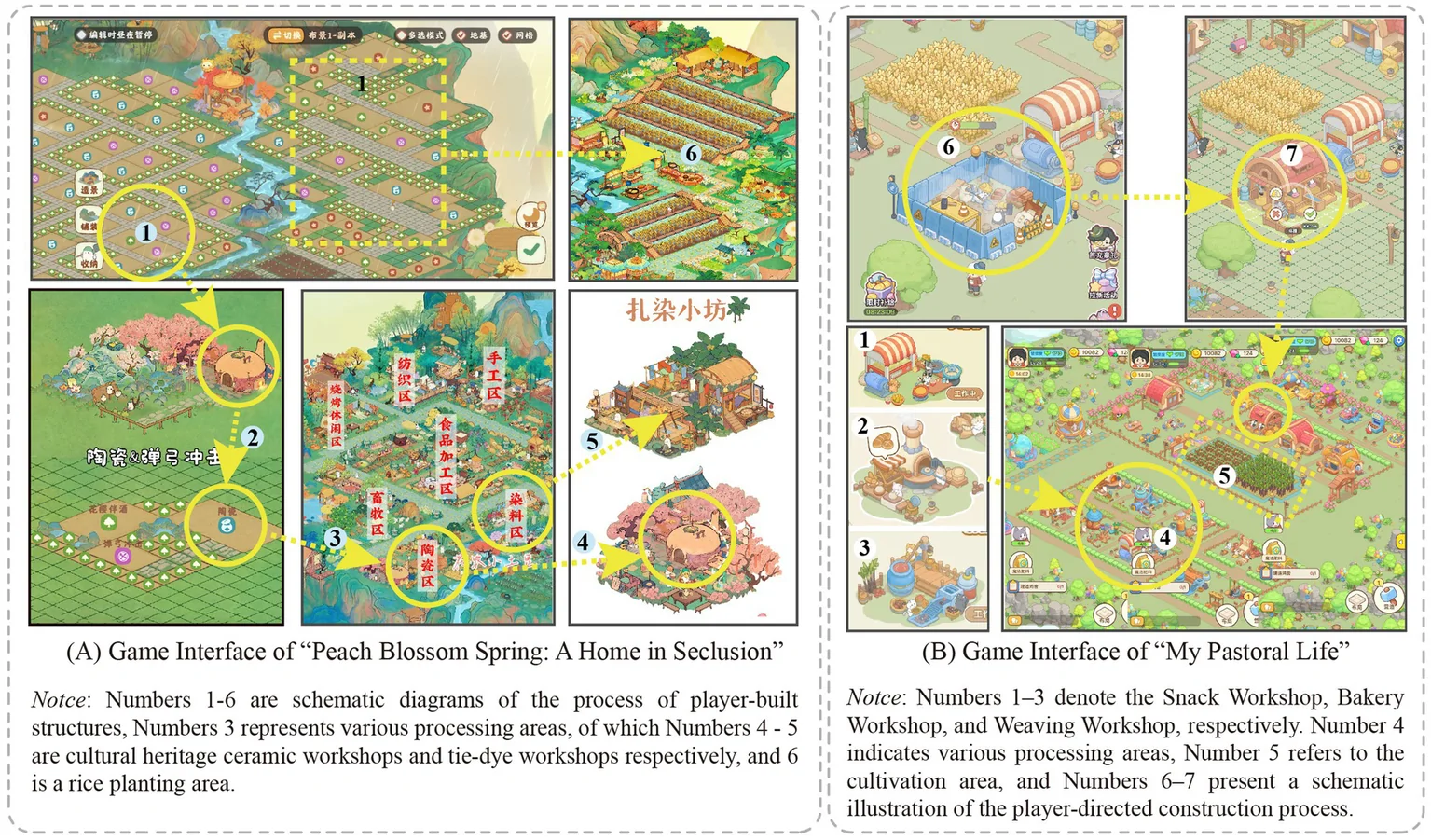
Game interface.

As shown in Figure 2A, this study uses Taoyuan: Deep in the Village, a management-style Serious Game with salient Intangible Cultural Heritage (ICH) embedding, as the experimental stimulus. Set in a narrative world of traditional rural life and handicraft practice, the game combines Chinese ink-painting aesthetics, pastoral spatial structures, and gameplay centered on sustainable management and courtyard construction. Its distinctive value lies not merely in entertainment, but in constructing a culturally embedded digital experiential environment through heritage narratives, authenticity cues, and autonomous construction. Contextualized storytelling, localized social relations, ICH-related tasks, and everyday rural elements support Heritage Narrative Involvement; architectural forms, environmental imagery, craft symbols, object details, and visual aesthetics strengthen the perception of the virtual heritage space as authentic, credible, and culturally grounded; and players’ freedom to construct, arrange, and manage courtyards at their own pace makes autonomous participation and self-expression central to gameplay. Together, these features provide the psychological basis for the emergence of Cultural Identity, Virtual Heritage Place Attachment, and Flow. Group B served as a non-heritage Serious Game active control (My Ranch Life; see Figure 2B), matched on gameplay structure but excluding Intangible Cultural Heritage (ICH) content, heritage narratives, and culturally meaningful space. Relative to the experimental condition, it exhibited lower cultural meaning density, emphasized functional representation over Perceived Authenticity, and supported task-based rather than culturally grounded autonomous construction, thereby isolating the additional effects of heritage narratives, Perceived Authenticity, and heritage-based spatial construction beyond general Serious Game mechanisms.

To minimize differences unrelated to cultural content, the two game conditions were matched at the design and procedural levels. Both games adopted a management-style gameplay structure involving spatial arrangement, resource-related tasks, goal-directed progression, and repeated interaction with the game environment. The two conditions were also controlled in terms of gameplay duration, device format, experimental setting, task instructions, and pretest–posttest procedure. The active-control condition was therefore not designed as a passive or low-quality comparison condition, but as a non-heritage management-game condition that retained general gameplay participation while minimizing explicit heritage narratives, ICH-related content, heritage symbols, and culturally meaningful spatial construction. The matching strategy is summarized in Supplementary Table 1.

### 3.3 Study 1: randomized active-control experiment

#### 3.3.1 Experimental purpose and methodological positioning

The first study uses a pretest–intervention–posttest AB-group randomized active-controlled design to test the immediate regulatory effect of heritage-based Serious Games on university students’ State Anxiety under high experimental control. Relative to cross-sectional surveys, this design is better suited to detecting short-term changes in State Anxiety and supporting causal inference. Its purpose is not to test the full theoretical model, but to address the more basic question of whether culturally embedded Serious Games reduce State Anxiety more effectively than the active control condition. To ensure manipulation validity and internal validity, a pilot study was conducted before the formal experiment to calibrate the stimulus materials and verify between-group differences in Heritage Narrative Involvement, Perceived Authenticity, and Perceived Autonomy, while controlling for playability, interface clarity, and operational load. Study 1 therefore serves primarily as effect identification, whereas the later studies are designed to explain why the effect emerges.

#### 3.3.2 Participants

The sample comprised 130 participants, all of whom were undergraduate or master’s students approaching graduation. Following screening for questionnaire completeness and response validity, all 130 cases were retained. As reported in Supplementary Table 2, gender distribution was relatively balanced, with 58 males (44.62%) and 72 females (55.38%). Participants were predominantly aged 21–27 years: 32 were aged 21–22 (24.62%), 61 were aged 23–24 (46.92%), 27 were aged 25–26 (20.77%), and 10 were aged 27 or above (7.69%). Educationally, 78 participants were undergraduates (60.00%) and 52 were master’s students (40.00%). These demographic characteristics are broadly consistent with the target population of young adults in the graduation transition period, supporting the suitability of the sample for subsequent empirical analysis.

#### 3.3.3 Experimental procedure

A pilot study was conducted to establish between-group differentiation in Heritage Narrative Involvement, Perceived Authenticity, and Perceived Autonomy, while ensuring comparability in playability, interface clarity, and operational load. The active control condition (Group B) retained the core gameplay mechanics but reduced cultural embedding and heritage narrative content; minor adjustments were implemented to enhance internal validity and experimental comparability. The formal experiment adopted a pretest–intervention–posttest design. After providing electronic informed consent, participants completed the pretest questionnaire (T0), which assessed baseline State Anxiety and relevant control variables. State Anxiety was measured using the State Anxiety Inventory (STAI-S), a 20-item instrument rated on a 4-point Likert scale, with total scores ranging from 20 to 80, where higher scores indicate greater anxiety (Shain et al., 2020; Spielberger et al., 1983). Participants were then randomly assigned to either the experimental or active control condition and engaged in a single ~30-min gameplay session on a standardized mobile device in a quiet environment. Immediately following the intervention, participants completed the posttest questionnaire (T1; see Supplementary Table 3), which assessed manipulation checks, key mediating variables, and the outcome measure. Comparisons between T0 and T1 across conditions were used to estimate the immediate regulatory effect of heritage-based serious games on State Anxiety and to provide initial evidence for the proposed psychological mechanisms.

### 3.4 Study 2: quantitative phase

#### 3.4.1 Measurement instrument

The questionnaire was developed using established scale-development procedures. Items were adapted from prior literature to fit the digital heritage context, generating an initial draft. To establish content validity, five domain experts reviewed the instrument with respect to wording, logical coherence, and alignment with the research objectives, and their feedback guided subsequent revision. The research team then finalized the pilot version. All constructs were measured on a 7-point Likert scale (1 = “strongly disagree,” 7 = “strongly agree”), and respondents were instructed to indicate the option that best reflected their actual experience. This measurement design was intended to support data quality and analytical feasibility. The full set of items is presented in Table 1.

**Table 1:** Demographic characteristics of respondents (*N* = 130).

Variable	Category	Frequency	Percentage
Gender	Male	58	44.62%
	Female	72	55.38%
Age	21–22	32	24.62%
	23–24	61	46.92%
	25–26	27	20.77%
	≥27	10	7.69%
Education	Undergraduate	78	60.00%
	Master’s	52	40.00%
Total		130	100%

#### 3.4.2 Pre-research analysis

In Study 2, a questionnaire survey tested the proposed cultural–psychological mechanism model. Because the core variables—Heritage Narrative Involvement, Perceived Authenticity, Perceived Autonomy, Cultural Identity, Virtual Heritage Place Attachment, Flow, and State Anxiety—represent post-gameplay subjective perceptions, primary data were collected using structured measurement scales for PLS-SEM and ANN analyses. A pilot study in February 2026 (*N* = 30) assessed item clarity and feasibility, leading to minor revisions. Cronbach’s *α* and composite reliability met recommended thresholds, indicating acceptable internal consistency and supporting large-sample validation in Study 2.

#### 3.4.3 Data collection procedure

Primary data were collected through a structured online questionnaire targeting users with prior experience playing Taoyuan: Deep in the Village. The survey was disseminated through game communities, social media platforms, and interest groups related to digital games and cultural heritage. Before participation, respondents were informed that the study was for academic purposes only, that all data would remain anonymous and confidential, and that participation was voluntary with informed consent obtained. In total, 529 questionnaires were distributed. Data quality screening followed predefined criteria: cases with incomplete or substantial missing data were removed; responses with abnormally short completion times were excluded; straight-lining or patterned responses on Likert-type items were discarded; and cases failing attention or consistency checks were eliminated. After screening, 473 valid responses were retained (effective response rate = 89.4%). This sample size is adequate for PLS-SEM estimation and sufficient to support subsequent ANN-based nonlinear predictive analysis.

#### 3.4.4 Participant profile

A total of 473 valid questionnaires were retained for the final analysis. As reported in Supplementary Table 4, gender distribution was relatively balanced, with 250 males (52.85%) and 223 females (47.15%). The sample was predominantly young, including 193 respondents aged 18–22 (40.80%), 157 aged 23–26 (33.19%), 79 aged 27–29 (16.70%), and 44 in other age groups (9.30%). Educationally, 194 respondents held a bachelor’s degree (41.01%), 118 a master’s degree (24.95%), and 49 a doctoral degree (10.36%), while 112 (23.68%) were faculty members or working professionals. These characteristics indicate that the sample is broadly consistent with the target population and thus suitable for subsequent empirical analysis.

### 3.5 Study 3: qualitative phase

To further interpret the quantitative findings from the first two studies, a qualitative investigation was conducted in Study 3. Whereas Study 1 identifies effects and Study 2 tests structural pathways, Study 3 focuses on how key mechanisms are formed from the perspective of players’ lived gameplay experience. It examines how players interpret heritage narratives and cultural cues, perceive authenticity and autonomy, internalize cultural content as self-relevant experience, and in turn develop Virtual Heritage Place Attachment, Flow, and emotional relief. Study 3 thus provides experiential-level accounts that complement the pathways identified in the first two studies, strengthening both the psychological interpretation of the mechanisms and the ecological validity of the findings.

#### 3.5.1 Procedure

To reduce recall bias, only participants who could clearly recall their gameplay experiences and related questionnaire responses were included in Study 3. Interview invitations were distributed via WeChat together with a study description and informed consent form. All interviews were conducted in person as semi-structured sessions in quiet, private settings and lasted approximately 25–30 min. Eligible participants were required to satisfy the basic screening criteria used in the first two studies and to provide clear verbal accounts of their gameplay experiences. The interview guide focused on the core constructs identified in the earlier studies, including Heritage Narrative Involvement, Perceived Authenticity, Perceived Autonomy, Cultural Identity, emotional attachment to virtual heritage spaces, Flow, and experiences of anxiety reduction. With participant consent, all interviews were audio-recorded and transcribed verbatim. The transcripts were analyzed using grounded theory procedures—open coding, axial coding, and selective coding—to identify themes that further explained and contextualized the quantitative findings. Two researchers independently coded an initial subset of transcripts to develop a preliminary codebook. Coding discrepancies were discussed until consensus was reached, and the codebook was then refined and applied to the remaining transcripts. During axial coding, conceptually similar codes were grouped into categories, and during selective coding, these categories were integrated around the core explanatory process. To enhance analytical rigor, the research team conducted repeated comparisons between raw transcripts, codes, categories, and the theoretical model. Six additional interviews were reserved for theoretical saturation testing, and no new core categories or category relationships emerged. The coding process is illustrated in Supplementary Figure 2.

#### 3.5.2 Sample

Study 3 used purposive sampling to recruit follow-up interview participants from those who had previously completed Study 1. In total, 33 individuals were contacted, and five were excluded because they were unable to clearly recall their earlier questionnaire responses. The final qualitative sample comprised 28 participants. The sample included doctoral, master’s, and undergraduate students, ensuring a degree of cross-disciplinary and cross-contextual heterogeneity. Participants were also active across major digital platforms, including Xiaohongshu, Douyin, gaming communities, and gaming websites, indicating diverse digital media use contexts and varying levels of exposure to conversational artificial intelligence. Participant demographics are reported in Supplementary Table 5.

#### 3.5.3 Semi-structured interview questions

The semi-structured interview protocol was developed to clarify the key mechanisms identified in Study 1 and Study 2 rather than to explore issues unrelated to gameplay experience. The interview questions were therefore organized around the core constructs of the research model: Heritage Narrative Involvement, Perceived Authenticity, Perceived Autonomy, Cultural Identity, Virtual Heritage Place Attachment, Flow, and State Anxiety reduction. The interviews focused on how players interpreted heritage narratives and cultural content, evaluated the authenticity of game environments, cultural symbols, and interaction forms, experienced autonomy in spatial construction and management, and how these processes contributed to Cultural Identity, Virtual Heritage Place Attachment, immersive engagement, and short-term emotional relief. This design retained the openness of qualitative inquiry while ensuring strong conceptual alignment with the quantitative findings from the first two studies. The interview questions are reported in Supplementary Table 6.

## 4 Study 1: effect identification results

### 4.1 Preliminary analysis and baseline equivalence testing

Prior to testing the intervention effects, baseline comparability between the experimental group and the active control group was examined. The results indicate that there were no significant differences between the two groups in terms of gender, age distribution, or educational level (see Table 2), suggesting good comparability in demographic characteristics. Further independent-samples t-tests show that the two groups did not differ significantly in prior gaming experience, cultural familiarity, cultural interest, or baseline State Anxiety (all *p* > 0.05).

**Table 2:** Behavioral measurement scale.

Variables	Code	Content	Original reference
Heritage narrative involvement	HNI	The intangible cultural heritage narrative in the game kept me wanting to know what would happen next.	Rizvic et al. (2024)
		During the experience, I naturally focused my attention on the cultural stories presented in the game.	
		The game’s traditional story and character relationships gave me a strong sense of immersion.	
Perceived authenticity	PA	I believe the cultural details in the game have credible traditional cultural basis.	Zhu et al. (2025)
		The intangible cultural heritage elements in the game made me feel that they had real cultural significance, rather than just being decorations.	Bryce et al. (2015)
		Overall, I felt that the game created a realistic and believable atmosphere of heritage.	
Cultural identity	CI	After playing this game, I am very satisfied with the traditional culture it presents.	Wang and Meng (2024)
		The cultural content in the game made me feel that it was connected to my cultural identity.	Yang and Li (2025)
		The intangible cultural heritage in the game made me feel that it was more relevant to the cultural values I identify with.	
		This game strengthened my psychological connection with the relevant traditional cultural significance.	
Perceived autonomy	PAU	During the game, I could decide how to build and arrange the space according to my own ideas.	Ballou et al. (2024)
		I feel that I can advance the game experience in a way that I approve of.	
		The game gave me enough freedom of choice, rather than forcing me to follow a single path.	
		While playing this game, I felt that my actions were in my own hands.	
Virtual heritage place attachment	VHPA	I gradually developed an emotional attachment to this courtyard/village in the game.	Chan et al. (2024)
		This virtual heritage space gives me a sense of “this is my place.”	Deng et al. (2026)
		This virtual heritage space holds special local significance for me.	Qiu et al. (2025)
Flow	FL	When playing this game, I often become completely absorbed in the current activity.	Oliveira and Hamari (2025)
		During the experience, I sometimes forgot about my surroundings or the passage of time.	
		I felt highly integrated into the ongoing activity within the game.	
		The entire experience kept me in a state of continuous engagement, smooth flow, and immersion.	
State anxiety	SA	After playing this game, I feel calm now.	Valente et al. (2025)
		After playing this game, I feel relaxed right now.	
		After playing this game, I feel very comfortable right now.	
		After playing this game, I feel relaxed right now.	
		After playing this game, my anxiety was reduced.	

### 4.2 Manipulation check results

Study 1 examined whether the heritage-based Serious Game successfully activated the targeted culturally embedded stimulus dimensions. Posttest comparisons showed that the experimental group scored significantly higher than the active control group on Heritage Narrative Involvement, *t*(128) = 5.65, *p* < 0.001, Cohen’s *d* = 0.99, *M* = 4.18, SD = 0.63 vs. M = 3.46, SD = 0.81; Perceived Authenticity, *t*(128) = 5.06, *p* < 0.001, Cohen’s *d* = 0.89, *M* = 4.11, SD = 0.59 vs. *M* = 3.52, SD = 0.73; and Perceived Autonomy, *t*(128) = 2.97, *p* = 0.004, Cohen’s *d* = 0.52, *M* = 4.06, SD = 0.56 vs. *M* = 3.74, SD = 0.67 (see Table 3). These results indicate successful manipulation of Heritage Narrative Involvement, Perceived Authenticity, and Perceived Autonomy. The moderate effect size for Perceived Autonomy further suggests that the active control remained basically playable and engaging rather than constituting a low-quality or negative-experience condition. Overall, the results support the construct validity of the stimulus design and justify the subsequent main-effect analyses.

**Table 3:** Participants’ demographic information (*n* = 473).

Attribute	Items	Numbers	Percentage (%)
Gender	Male	268	52.86
	Female	239	47.14
Age	18–22	195	38.46
	23–26	168	33.14
	27–29	97	19.13
	Other age groups	47	9.27
Education background	Bachelor’s Degree	208	41.03
	Master’s Degree	127	25.05
	Doctoral Degree	52	10.26
	Staff	120	23.67
All	–	507	100

### 4.3 Main intervention effects on state anxiety

Study 1 examined whether heritage-based Serious Games reduced State Anxiety more effectively than the active control condition after short-term gameplay. A 2 (Group) × 2 (Time) mixed-design ANOVA on State Anxiety Inventory (SAI) scores showed a significant main effect of Time, *F*(1, 128) = 45.67, *p* < 0.001, partial η^2^ = 0.263, no main effect of Group, *F*(1, 128) = 0.30, *p* = 0.586, partial η^2^ = 0.002, and a significant Group × Time interaction, *F*(1, 128) = 8.31, *p* = 0.005, partial η^2^ = 0.061, indicating differential pretest–posttest change between groups (see Table 4). The experimental group declined from 49.88 (SD = 7.45) to 40.77 (SD = 7.87), whereas the active control group declined from 49.29 (SD = 8.02) to 45.63 (SD = 8.14). Paired-samples t tests confirmed significant reductions in both groups, with a larger decrease in the experimental group (*t* = 7.07, *p* < 0.001) than in the active control group (*t* = 2.65, *p* = 0.010; see Table 5). Overall, these results indicate that heritage-based Serious Games produce stronger short-term reductions in State Anxiety than general gameplay, providing the basis for subsequent mechanism analysis.

**Table 4:** Baseline equivalence test of demographic variables between the experimental group and the active control group (*N* = 130).

Variables	Category	Experimental group A (*n* = 65)	Control group B (*n* = 65)	*χ* ^2^	*P*
Gender	Male	27	31	0.28	0.597
	Female	38	34		
Age_Group	21–22	15	17	0.31	0.958
	23–24	32	29		
	25–26	13	14		
	≥27	5	5		
Education	Undergraduate	41	37	0.29	0.591
	Master’s	24	28		

**Table 5:** Baseline equivalence test of continuous variables between the experimental group and the active control group.

Variables	Experimental group A	Control group B	*t*	*P*
	(M ± SD)	(M ± SD)		
Game_Experience	3.10 ± 0.80	3.00 ± 0.79	0.71	0.477
Culture_Familiarity	3.00 ± 0.70	3.00 ± 0.70	0.01	0.996
Culture_Interest	3.40 ± 0.70	3.30 ± 0.70	0.81	0.419
SAI*T0*Total	49.88 ± 7.45	49.29 ± 8.02	0.43	0.667

## 5 Study 2: quantitative study results

### 5.1 PLS-SEM results

Following established PLS-SEM guidance (Hair et al., 2017; Leguina, 2015; Sarstedt et al., 2021), a two-step modeling approach was implemented using SmartPLS 4.1 (Anderson and Gerbing, 1988). The measurement model was first assessed using the PLS algorithm, and the research hypotheses were subsequently evaluated using a bootstrapping procedure.

#### 5.1.1 Assessment of measurement model

Measurement model quality was evaluated using Cronbach’s α, composite reliability, and average variance extracted, consistent with established psychometric principles concerning reliability and construct validity (Kline, 1999), and Average Variance Extracted (AVE). Values above 0.70 for Cronbach’s α and CR indicate acceptable internal consistency (Nunnally and Bernstein, 1994). As reported in Table 6, Cronbach’s α ranged from 0.878 to 0.941, CR from 0.879 to 0.945, and AVE from 0.784 to 0.873 across all latent constructs. These values all exceeded recommended thresholds, supporting internal consistency reliability and convergent validity (Fornell and Larcker, 1981). Multicollinearity was assessed using the variance inflation factor (VIF), with its interpretation guided by established treatments of applied linear statistical models (Neter et al., 1985). Nearly all item-level VIF values were below 5; although PAU2 (5.096) and PAU3 (5.149) slightly exceeded this criterion, the results do not indicate a serious multicollinearity problem.

**Table 6:** Cronbach’s α, corporate reliability and average variance extracted.

Variables	Cronbach’s alpha	Composite reliability	AVE	VIF	Items	Factor loading
C I	0.920	0.924	0.807	2.651	CI1	0.877
				4.204	CI2	0.927
				4.326	CI3	0.926
				2.752	CI4	0.862
FL	0.912	0.913	0.792	2.548	FL1	0.868
				3.282	FL2	0.901
				3.570	FL3	0.912
				2.722	FL4	0.878
HNI	0.928	0.933	0.873	3.460	HNI1	0.918
				4.846	HNI2	0.954
				3.454	HNI3	0.931
PA	0.898	0.904	0.831	2.967	PA1	0.922
				3.295	PA2	0.926
				2.397	PA3	0.886
PAU	0.941	0.945	0.850	3.902	PAU1	0.903
				5.096	PAU2	0.936
				5.149	PAU3	0.945
				3.460	PAU4	0.902
SA	0.887	0.914	0.784	1.995	SA1	0.842
				2.289	SA2	0.840
				2.493	SA3	0.858
				2.769	SA4	0.826
				2.448	SA5	0.768
VHPA	0.878	0.879	0.803	2.522	VHPA1	0.887
				3.179	VHPA2	0.921
				2.127	VHPA3	0.880

In addition, measurement validity was evaluated in terms of convergent and discriminant validity. Convergent validity was supported by factor loadings and AVE: all item loadings exceeded 0.70, and AVE values ranged from 0.784 to 0.873, all above the recommended 0.50 threshold, indicating satisfactory convergent validity (see Table 7). Discriminant validity was assessed using the Fornell–Larcker criterion. As shown in Table 8, the square root of each construct’s AVE (diagonal elements) exceeded its correlations with other constructs, supporting adequate discriminant validity (Fornell and Larcker, 1981).

**Table 7:** Results of mixed-design ANOVA for state anxiety.

Effect	*F*	*p*	Partial η^2^
Group	0.3	0. 586	0.002
Time	45.67	<0.001	0.263
Group × Time	8.31	0.005	0.061

**Table 8:** Cronbach’s α, corporate reliability and average variance extracted.

Variables	Cronbach’s alpha	Composite reliability	AVE	VIF	Items	Factor loading
C I	0.92	0.924	0.807	2.651	CI1	0.877
				4.204	CI2	0.927
				4.326	CI3	0.926
				2.752	CI4	0.862
FL	0.912	0.913	0.792	2.548	FL1	0.868
				3.282	FL2	0.901
				3.57	FL3	0.912
				2.722	FL4	0.878
HNI	0.928	0.933	0.873	3.46	HNI1	0.918
				4.846	HNI2	0.954
				3.454	HNI3	0.931
PA	0.898	0.904	0.831	2.967	PA1	0.922
				3.295	PA2	0.926
				2.397	PA3	0.886
PAU	0.941	0.945	0.85	3.902	PAU1	0.903
				5.096	PAU2	0.936
				5.149	PAU3	0.945
				3.46	PAU4	0.902
SA	0.887	0.914	0.784	1.995	SA1	0.842
				2.289	SA2	0.84
				2.493	SA3	0.858
				2.769	SA4	0.826
				2.448	SA5	0.768
VHPA	0.878	0.879	0.803	2.522	VHPA1	0.887
				3.179	VHPA2	0.921
				2.127	VHPA3	0.88

#### 5.1.2 Assessment of structural model

The structural model was assessed using path coefficients, the coefficient of determination (R^2^), and predictive relevance (Q^2^). Path significance was estimated in SmartPLS 4.1 using bootstrapping with 5,000 resamples. As reported in Tables 8, 9 hypothesized paths were tested; 9 were supported and 1 was not. At the stimulus-to-organism level, Heritage Narrative Involvement positively predicted Cultural Identity (HNI → CI: *β* = 0.181, t = 4.244, *p* < 0.001). Perceived Authenticity positively predicted both Cultural Identity and Virtual Heritage Place Attachment (PA → CI: *β* = 0.248, *t* = 5.821, *p* < 0.001; PA → VHPA: *β* = 0.233, *t* = 6.090, *p* < 0.001). Perceived Autonomy likewise positively predicted Cultural Identity, Flow, and Virtual Heritage Place Attachment (PAU → CI: *β* = 0.187, *t* = 4.430, *p* < 0.001; PAU → FL: *β* = 0.204, *t* = 4.386, *p* < 0.001; PAU → VHPA: *β* = 0.118, *t* = 2.859, *p* = 0.004). Within the organism layer, Cultural Identity positively predicted Virtual Heritage Place Attachment (CI → VHPA: *β* = 0.325, *t* = 7.174, *p* < 0.001), whereas the path from Virtual Heritage Place Attachment to Flow was not significant (VHPA → FL: *β* = 0.081, *t* = 1.709, *p* = 0.087). At the organism-to-response level, both Flow and Virtual Heritage Place Attachment significantly predicted State Anxiety (FL → SA: *β* = 0.122, *t* = 2.843, *p* = 0.004; VHPA → SA: *β* = 0.278, *t* = 7.367, *p* < 0.001). Taken together, the findings suggest that Heritage Narrative Involvement, Perceived Authenticity, and Perceived Autonomy affect State Anxiety indirectly through Cultural Identity, Virtual Heritage Place Attachment, and Flow, with the sole unsupported path being VHPA → FL.

**Table 9:** Descriptive statistics of changes in state anxiety (T0–T1) for Groups A and B.

Group	T0	T1	Average change	Paired t	*p*
	(M ± SD)	(M ± SD)	(T1–T0)		
Experimental Group A	49.88 ± 7.45	40.77 ± 7.87	−9.11	7.07	< 0.001
Active control Group B	49.29 ± 8.02	45.63 ± 8.14	−3.66	2.65	0.01

**Figure 3:**
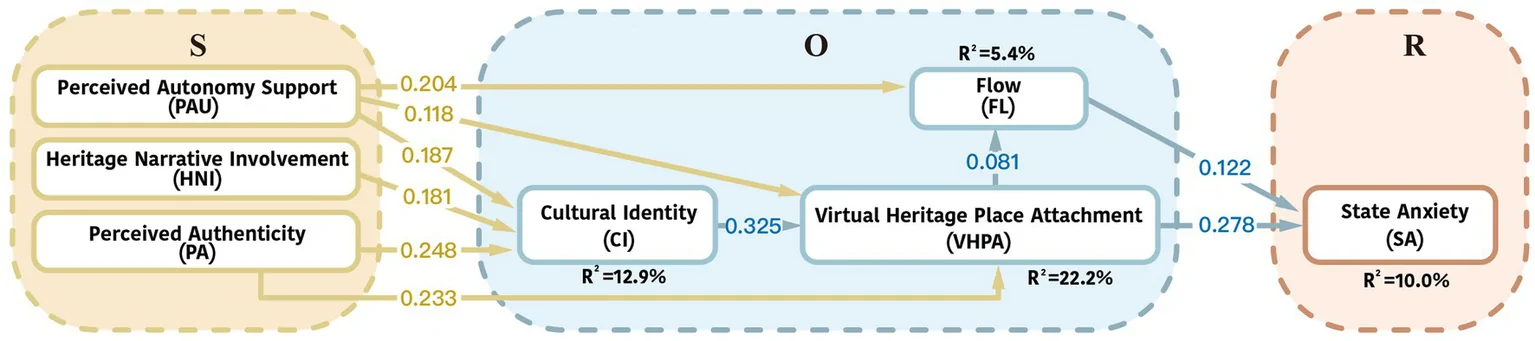
PLS results of the structural model.

As shown in Figure 3, the model explains the variance in Cultural Identity (*R*^2^ = 12.9%), Virtual Heritage Place Attachment (*R*^2^ = 22.2%), Flow (*R*^2^ = 5.4%), and State Anxiety (*R*^2^ = 10.0%). Furthermore, the predictive relevance of the model for the four endogenous constructs was assessed. The *Q*^2^ values for Cultural Identity (*Q*^2^ = 0.119), Virtual Heritage Place Attachment (*Q*^2^ = 0.204), Flow (*Q*^2^ = 0.041), and State Anxiety (*Q*^2^ = 0.085) are all greater than zero, indicating that the model demonstrates adequate predictive capability (Sarstedt et al., 2021).

### 5.2 ANN results

#### 5.2.1 Model development

**Figure 4:**
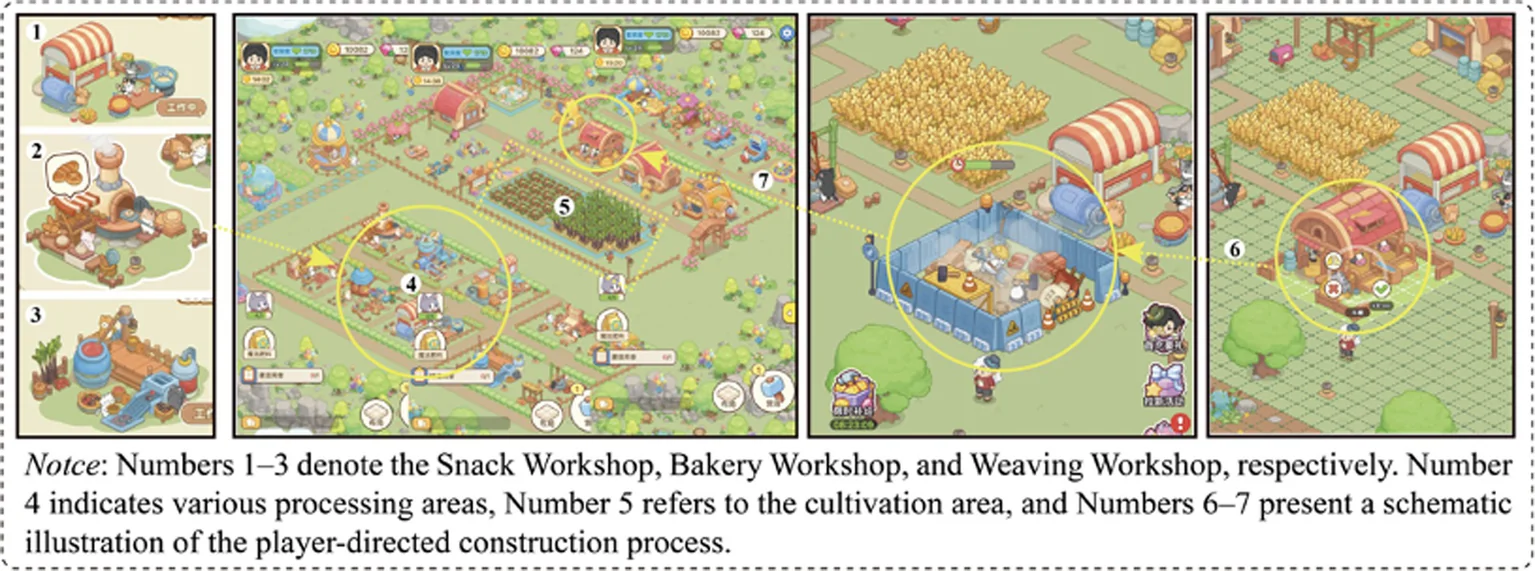
Game interface of *“My Pastoral Life.”*

Because Partial Least Squares Structural Equation Modeling (PLS-SEM) is primarily suited to modeling linear and compensatory relationships among latent variables (Lo et al., 2022), it may not fully capture potential nonlinear effects. To address this limitation, Study 2 incorporated Artificial Neural Networks (ANN) as a complementary analytic approach for nonlinear prediction. Following Chong (2013), four ANN models were specified (see Supplementary Figure 3) on the basis of the SEM-supported paths (see Figure 4) to predict four endogenous variables: Cultural Identity (CI), Virtual Heritage Place Attachment (VHPA), Flow (FL), and State Anxiety (SA). Model A used Heritage Narrative Involvement (HNI), Perceived Authenticity (PA), and Perceived Autonomy (PAU) as inputs to predict CI; Model B used CI, PA, and PAU to predict VHPA; Model C used VHPA and PAU to predict FL; and Model D used FL and VHPA to predict SA. All models were estimated in SPSS 27.0 with a single-hidden-layer architecture. To minimize random-initialization bias, each model was trained ten times. Performance was assessed using Root Mean Square Error (RMSE), and sensitivity analysis was used to estimate predictor importance.

#### 5.2.2 Validation of ANN

To minimize overfitting, the Artificial Neural Network (ANN) models were evaluated using 10-fold cross-validation, with 90% of cases used for training and 10% for testing (Xie and Wang, 2025). As reported in Table 10, four ANN models were estimated to predict Cultural Identity (CI), Virtual Heritage Place Attachment (VHPA), Flow (FL), and State Anxiety (SA). Across ten repeated runs, Root Mean Square Error (RMSE) values for both training and testing were within acceptable ranges for all models. Mean training RMSE values for Models A–D were 0.447, 0.399, 0.487, and 0.461, respectively, whereas mean testing RMSE values were 0.410, 0.369, 0.450, and 0.425. Model B yielded the lowest prediction error, suggesting that the model using CI, PAU, and PA to predict VHPA showed the strongest predictive stability. By contrast, Models C and D produced higher mean testing RMSE values (0.450 and 0.425), indicating greater predictive difficulty for FL from VHPA and PAU, and for SA from FL and VHPA. Training-phase variability was low across all models (SD = 0.007–0.017), indicating stable estimation, whereas testing-phase variability was higher, especially for Model C (SD = 0.110) and Model D (SD = 0.118), suggesting weaker generalization stability. Overall, all four ANN models showed acceptable predictive performance, although Model B performed best and Models C and D were comparatively less stable at test study.

**Table 10:** ANCOVA results for posttest (T1) state anxiety after controlling for covariates.

Source	*F*	*p*
Group	12.69	<0.001
SAIT0Total	0.46	0.5
Game_Experience	3.9	0.051
Culture_Familiarity	0.57	0.453
Culture_Interest	0.52	0.474

Model fit was further assessed using the coefficient of determination (*R*^2^), calculated according to Equations 1, 2. The ANN models explained 57.99% of the variance in Cultural Identity (CI), 59.89% in Virtual Heritage Place Attachment (VHPA), 49.85% in Flow (FL), and 35.89% in State Anxiety (SA), indicating generally satisfactory fit and generalization performance.


$$\text{RMSE}=\sqrt{\mathrm{MSE}}=\frac{\mathrm{SSE}}{n}$$ (1)

$$R^{2}=1-\frac{\mathrm{SSE}}{\mathrm{SST}}$$ (2)

In Equation 1, MSE denotes the mean squared error, SSE represents the sum of squared errors, and n refers to the sample size of the training and testing datasets.

#### 5.2.3 Sensitivity analysis

Permutation-based sensitivity analysis was conducted to examine the Artificial Neural Network (ANN) models and estimate the relative importance of each predictor for the endogenous variables (Roy et al., 2023). As reported in Table 8, for Model A (output: CI), normalized importance was ranked as PA (100.000%) > PAU (86.988%) > HNI (54.217%), indicating that Perceived Authenticity was the strongest predictor of Cultural Identity, followed by Perceived Autonomy, whereas Heritage Narrative Involvement was less influential. For Model B (output: VHPA), the ranking was PA (100.000%) > CI (96.212%) > PAU (56.313%), suggesting that Perceived Authenticity contributed most to Virtual Heritage Place Attachment, followed by Cultural Identity, with Perceived Autonomy contributing least. For Model C (output: FL), PAU (100.000%) exceeded VHPA (56.986%), indicating that Perceived Autonomy was the dominant predictor of Flow. For Model D (output: SA), VHPA (100.000%) exceeded FL (76.014%), showing that Virtual Heritage Place Attachment was the strongest predictor of State Anxiety, whereas Flow played a secondary role. To compare PLS-SEM and ANN, the study further juxtaposed the ranking of SEM path coefficients with the ranking of normalized importance values from ANN sensitivity analysis (see Table 11). The two methods yielded fully consistent rankings in Models A, C, and D. A minor discrepancy appeared only in Model B: PLS-SEM indicated that CI → VHPA was slightly stronger than PA → VHPA, whereas ANN suggested that PA had marginally greater predictive importance than CI. Despite this difference, both methods converged in identifying Perceived Authenticity and Cultural Identity as the primary antecedents of Virtual Heritage Place Attachment, with Perceived Autonomy making the weakest contribution. Overall, the ANN results corroborate the main PLS-SEM findings while adding information about the relative predictive strength of antecedent variables, thereby enhancing the robustness and explanatory depth of the study.

**Table 11:** Fornell-Larcker criterion.

CI	FL	HNI	PA	PAS	SA	VHPA	
CI	0.898						
FL	−0.004	0.89					
HNI	0.188	0.085	0.935				
PA	0.239	−0.058	−0.015	0.912			
PAS	0.19	0.218	0.059	−0.031	0.922		
SA	0.136	0.155	0.09	0.025	0.03	0.827	
VHPA	0.403	0.116	0.074	0.306	0.173	0.292	0.896

## 6 Study 3: qualitative study results

### 6.1 Grounded theory approach

All interviews were audio-recorded and transcribed verbatim. The 28 transcripts were analyzed using grounded theory procedures—open coding, axial coding, and selective coding—within a constant comparative framework. Initial concepts were derived from participants’ accounts of gameplay experience, grouped into higher-order axial categories based on conceptual similarity and logical association, and then integrated into selective themes explaining how heritage-based Serious Games reduce State Anxiety. The resulting coding structure was closely aligned with the theoretical framework developed in Study 1 and Study 2, while extending the quantitative findings at the experiential level. The qualitative evidence suggests that emotional relief does not arise simply from generic relaxation or distraction, but through a progressive process of “cultural entry → authenticity validation → autonomous construction → identity and attachment formation → immersive engagement → emotional relief.” Study 3 therefore not only corroborates the relationships identified in the first two studies, but also clarifies how heritage-based Serious Games convert cultural content into a psychological resource for emotion regulation in lived experience. The grounded theory coding results are reported in Table 12.

**Table 12:** A summary of the results of hypotheses testing.

Hypothesis	Path coefficient	*p*-values	*T*-values	Original sample (O)	Sample mean (M)	Result
CI - > VHPA	0.325	0.000	7.174	0.325	0.324	Supported
FL - > SA	0.122*	0.004	2.843	0.122	0.124	Supported
HNI - > CI	0.181**	0.000	4.244	0.181	0.182	Supported
PA - > CI	0.248**	0.000	5.821	0.248	0.247	Supported
PA - > VHPA	0.233**	0.000	6.090	0.233	0.233	Supported
PAU - > CI	0.187**	0.000	4.430	0.187	0.188	Supported
PAU - > FL	0.204**	0.000	4.386	0.204	0.206	Supported
PAU - > VHPA	0.118*	0.004	2.859	0.118	0.118	Supported
VHPA - > FL	0.081	0.087	1.709	0.081	0.082	Not supported
VHPA - > SA	0.278**	0.000	7.367	0.278	0.281	Supported

** Indicates *p* < 0.001; * Indicates *p* < 0.01; and no asterisk indicates *p* < 0.05.

To evaluate the robustness of the category system, a theoretical saturation test was conducted using studied theoretical sampling and constant comparison. Of the 28 semi-structured interviews, the first 22 were used to establish the preliminary category structure through open, axial, and selective coding, while the remaining six were reserved for saturation testing. These six interviews were coded line by line to determine whether additional concepts, category properties, or inter-category relationships would emerge. The results indicated that the additional interviews primarily elaborated the existing framework by adding semantic detail and contextual variation related to Heritage Narrative Involvement, Perceived Authenticity, Cultural Identity, Virtual Heritage Place Attachment, and Flow formation. No new core categories emerged, and no existing category relationships were modified. The category system was therefore considered theoretically saturated within the scope of the current sample and study of analysis (Glaser and Strauss, 2017; Corbin and Strauss, 2014). At the same time, future research with more diverse samples, including different age groups, cultural backgrounds, or other heritage-game contexts, may identify additional complementary concepts. Overall, the saturation test enhances the credibility of the Study 3 findings and provides further support for the experiential processes underlying the mechanism pathways identified in Study 1 and Study 2.

### 6.2 Research findings

The qualitative findings indicate that the anxiety-reducing effects of heritage-based Serious Games cannot be explained solely by entertainment, distraction, or short-term relaxation. Instead, they appear to emerge through a progressive experiential sequence: cultural entry → authenticity validation → autonomous participation → identity and place-meaning formation → immersive engagement → emotional relief. Study 3 therefore not only corroborates the mechanism relationships identified in the first two studies, but also clarifies how these relationships are subjectively constituted in gameplay experience.

First, Heritage Narrative Involvement enabled participants to enter the cultural world of the game rather than merely recognize cultural symbols at a visual level. Several participants described the heritage narrative as a process of gradual cultural entry. For example, one participant stated: “Insert representative quote showing that the participant felt drawn into the heritage story or cultural context” (P01). This suggests that narrative involvement helped transform cultural content from external information into an experientially meaningful context. Second, Perceived Authenticity appeared to strengthen participants’ trust in and emotional response to the virtual heritage environment. Participants did not evaluate authenticity only in terms of visual realism; rather, they referred to the coherence of cultural symbols, spatial atmosphere, and traditional practices. One participant explained: “Insert representative quote showing that the participant perceived the game environment, symbols, or heritage elements as authentic, credible, or culturally grounded” (P07). Such responses indicate that authenticity validation allowed cultural content to enter deeper psychological processing. Third, Perceived Autonomy played an important role in transforming cultural experience into self-relevant participation. Participants frequently emphasized that constructing, arranging, and managing virtual spaces at their own pace made the cultural environment feel personally meaningful. As one participant noted: “Insert representative quote showing freedom of action, self-directed construction, or personal control in gameplay” (P12). This supports the interpretation that autonomy-supportive participation facilitated cultural internalization rather than passive reception. Fourth, through narrative involvement, authenticity validation, and autonomous participation, participants reported a growing sense of Cultural Identity and Virtual Heritage Place Attachment. Cultural content shifted from being merely perceived to being accepted, while the virtual environment gradually became a place associated with familiarity, belonging, and emotional meaning. One participant remarked: “Insert representative quote showing identification with cultural values, emotional connection with the virtual place, or a sense of belonging” (P16). This finding supports the view that cultural identity and place attachment are related expressions of cultural internalization and psychological ownership. Fifth, Flow emerged as a proximal experiential state, but the interviews suggest that it was not generated by place attachment alone. Participants associated flow more directly with focused attention, smooth interaction, self-paced gameplay, and temporary relief from distracting thoughts. For instance, one participant stated: “Insert representative quote showing concentration, immersion, loss of time awareness, or reduced distracting thoughts during gameplay” (P21). This helps explain why the quantitative path from Virtual Heritage Place Attachment to Flow was not significant: participants could experience the virtual heritage space as meaningful and emotionally familiar without necessarily entering a high-flow state. Finally, participants associated these cultural and experiential processes with reduced tension, worry, and emotional burden. One participant described this emotional change as follows: “Insert representative quote showing reduced anxiety, emotional calmness, relaxation, or relief after gameplay” (P25). Taken together, the qualitative findings support the central proposition that the emotional benefits of heritage-based Serious Games may depend substantially on the experiential internalization of cultural content as a psychological resource. At the same time, the interview evidence also refines the model by suggesting that Virtual Heritage Place Attachment and Flow may contribute to emotional relief through partly distinct routes: the former through meaning, belonging, and psychological settlement, and the latter through focused engagement, immersion, and reduced distracting processing.

## 7 Discussion

### 7.1 Key findings and responses to the research questions

This study moves beyond asking whether Serious Games reduce anxiety to examine whether heritage-based Serious Games operate through a distinct cultural–psychological mechanism beyond general game effects. Across the three studies, three main conclusions emerge. First, heritage-based Serious Games significantly reduced university students’ State Anxiety in the short term and did so more effectively than non-heritage Serious Games, thereby addressing RQ1. Second, this effect was not explained solely by gameplay participation, but was partly realized through internal psychological processes involving Cultural Identity, Virtual Heritage Place Attachment, and Flow, thereby addressing RQ2. Third, the findings generally supported a structured pathway from cultural internalization to place-based meaning and immersive engagement, although not all hypothesized links were confirmed; support for RQ3 should therefore be interpreted as conditional. Taken together, the results suggest that cultural content in digital experience can function not merely as contextual or aesthetic input, but as an active psychological resource with measurable emotional consequences. Heritage-based Serious Games should therefore be understood not simply as content carriers, but as culturally grounded experiential contexts with psychological consequences.

### 7.2 Relationship to prior research

A central finding is that heritage-based Serious Games may offer generate emotional benefits beyond general game effects, thereby extending previous research on Serious Games as anxiety interventions. Earlier studies have mainly explained anxiety reduction through generic processes such as distraction, relaxation, and immersion (Abd-Alrazaq et al., 2022; Schoneveld et al., 2018, 2020). The present findings suggest, however, that Serious Games are not psychologically equivalent, because their content structure may meaningfully shapes emotional outcomes. The active-control design helped reduce the possibility that the stronger effects observed in the experimental condition were driven solely by general gameplay participation. However, because full equivalence between the two games cannot be completely established, especially regarding unmeasured experiential dimensions such as engagement, enjoyment, immersion, and perceived difficulty, the between-condition difference should be interpreted as suggestive rather than conclusive evidence that cultural embedding played a contributing role. Thus, the findings support a culturally embedded interpretation of the anxiety-reduction effect, but do not establish that cultural embedding alone caused the observed difference. At the mechanism level, Heritage Narrative Involvement, Perceived Authenticity, and Perceived Autonomy jointly predicted Cultural Identity, consistent with prior work on narrative engagement (Guo et al., 2022; Rizvic et al., 2024), authenticity (Yi et al., 2024), and autonomy in Self-Determination Theory (Lüking et al., 2023; Yang et al., 2025). The contribution of the present study lies in integrating these factors into a unified mechanism, suggesting that cultural content is more likely to produce emotional effects when it is experienced as meaningful, credible, and self-relevant. The findings also extend Digital Heritage research by showing that the authenticity–place attachment relationship documented in prior work (Qiu et al., 2025; Zhu et al., 2025; Chan et al., 2024) can also emerge in interactive game contexts, where virtual heritage spaces become emotionally meaningful places through sustained interaction and self-directed construction. Finally, although Perceived Autonomy significantly predicted Flow, the path from Virtual Heritage Place Attachment to Flow was not significant. This nonsignificant result is theoretically informative rather than merely contradictory. Virtual Heritage Place Attachment primarily reflects an affective and cognitive bond with a culturally meaningful virtual place, whereas Flow is a more immediate experiential state characterized by focused attention, perceived control, task absorption, and ongoing interaction. In heritage-based Serious Games, attachment to a virtual heritage place may provide familiarity, emotional security, and place-based meaning, but these qualities do not necessarily translate into the intense attentional absorption required for Flow. Flow appears to depend more directly on autonomy-supportive gameplay, interactive control, feedback, and challenge–skill engagement than on place attachment alone. This interpretation is consistent with the finding that Perceived Autonomy significantly predicted Flow, whereas Virtual Heritage Place Attachment did not. Therefore, the nonsignificant VHPA → Flow path refines rather than weakens the model by indicating that place attachment may stabilize emotional experience, while Flow still depends on sustained engagement and perceived control.

### 7.3 Mechanism explanation and theoretical advancement

Overall, the findings support a cultural–psychological process involving cultural internalization, place-based meaning, and immersive engagement. Heritage Narrative Involvement, Perceived Authenticity, and Perceived Autonomy jointly transformed cultural content from external representation into self-relevant experience, leading to the emergence of Cultural Identity and its further development into Virtual Heritage Place Attachment. These constructs, together with Flow, functioned as proximal psychological mechanisms linking experience to emotional outcomes and jointly shaped changes in State Anxiety. However, the nonsignificant path from Virtual Heritage Place Attachment to Flow suggests that this mechanism should not be interpreted as a fully linear sequence in which place attachment necessarily produces immersive absorption. Rather, the findings indicate a more differentiated psychological process. Virtual Heritage Place Attachment and Flow appear to represent two related but partially independent proximal mechanisms. Virtual Heritage Place Attachment reflects the emotional anchoring of cultural meaning in a specific virtual heritage space, whereas Flow reflects an interaction-based experiential state supported by autonomy, perceived control, focused attention, and ongoing engagement. Therefore, attachment to a virtual heritage place may provide familiarity, emotional security, belonging, and place-based meaning, but it does not necessarily generate the intense attentional absorption required for Flow. Qualitative findings converged with these quantitative results by showing that anxiety reduction was experienced not as mere distraction, but as a process of cultural entry, authenticity validation, autonomous participation, and the gradual formation of identity and belonging. At the same time, the qualitative evidence also helps explain why the VHPA → Flow path was not significant. Participants could experience the virtual heritage space as meaningful, calming, or emotionally familiar without necessarily entering a strong Flow state. In other words, place attachment may contribute to emotional relief through meaning, belonging, and psychological settlement, whereas Flow may contribute through task-centered absorption, concentration, and reduced distracting processing. This convergence suggests that the emotional effects observed here arose from a coherent psychological pathway rather than from the additive influence of isolated mechanisms. The findings therefore support and extend the integration of the Stimulus–Organism–Response (SOR) framework and Self-Determination Theory (SDT): SOR captures the overall psychological process linking external experience to emotional outcomes, whereas SDT helps explain why autonomy facilitates the internalization of cultural content. More specifically, the present findings refine the organism layer of the SOR framework by showing that internal psychological responses do not necessarily form a strict serial chain. Cultural Identity and Virtual Heritage Place Attachment capture the meaning-based and place-based internalization of cultural content, whereas Flow captures the interaction-based immersive state. These mechanisms may converge on State Anxiety reduction through partly distinct routes. Thus, the unsupported VHPA → Flow path does not challenge the overall model; rather, it clarifies the boundary of the proposed mechanism and suggests that place-based meaning and immersive engagement should be treated as differentiated, complementary processes. Heritage-based Serious Games should thus be understood not as mere sources of stimulation, but as culturally grounded experiential contexts capable of eliciting internal psychological transformation.

At the same time, the present findings should not be interpreted as excluding other possible explanations for anxiety reduction. Nostalgia, emotional comfort, familiarity, escapism, and aesthetic pleasure may also contribute to the restorative effects of heritage-based Serious Games. For example, familiar cultural symbols and rural spatial atmospheres may evoke nostalgic feelings or a sense of emotional comfort; aesthetically pleasing visual design may induce positive affect and soft attentional engagement; and gameplay itself may provide temporary psychological distance from academic or social stressors. These processes are not necessarily inconsistent with the proposed cultural–psychological mechanism. Rather, they may operate as complementary affective pathways through which culturally embedded gameplay becomes emotionally restorative. However, because nostalgia, familiarity, escapism, emotional comfort, and aesthetic pleasure were not directly measured in this study, their independent or comparative contributions cannot be determined. The proposed model should therefore be understood as one theoretically specified pathway rather than an exhaustive explanation of anxiety reduction. Future research should explicitly measure these alternative mechanisms and examine whether they function as parallel mediators, antecedents of Virtual Heritage Place Attachment or Flow, or boundary conditions of the cultural–psychological process.

### 7.4 Cross-cultural transferability and local-culture adaptation

The present study was conducted in a Chinese cultural context and used a heritage-based Serious Game embedded in Chinese Intangible Cultural Heritage, rural spatial imagination, traditional aesthetics, and culturally meaningful construction practices. Therefore, the findings should not be interpreted as evidence that Chinese cultural content can be directly transferred to other countries to produce the same psychological effects. Rather, the cross-cultural implication of this study lies in the more general proposition that locally meaningful cultural resources may become psychological resources when they are experienced as authentic, self-relevant, and open to autonomous participation. From this perspective, the model may be transferable at the level of psychological process, but not necessarily at the level of cultural content. In other countries, the relevant cultural materials should be drawn from local heritage systems, collective memories, traditional narratives, spatial symbols, and community-based cultural practices. For example, a heritage-based Serious Game designed for another cultural context should not simply import Chinese symbols, but should translate the design logic of cultural narrative, authenticity, autonomy, place attachment, and immersion into locally meaningful forms. This means that local users, cultural experts, heritage practitioners, and community stakeholders should be involved in identifying which cultural elements are perceived as credible, emotionally meaningful, and personally relevant. At the same time, the psychological mechanisms may differ across cultures. In some cultural contexts, Cultural Identity may be the dominant pathway, especially when heritage content is closely connected to collective memory or national identity. In other contexts, nostalgia, familiarity, aesthetic pleasure, emotional comfort, or escapism may play a stronger role. The relative importance of Perceived Authenticity, Cultural Identity, Virtual Heritage Place Attachment, and Flow may also vary depending on users’ cultural familiarity, heritage ownership, ethnic background, migration experience, and prior exposure to the cultural content. Therefore, the present model should be regarded as a culturally situated but theoretically extendable framework, rather than a universally fixed mechanism. Future research should test this framework in different cultural settings using locally adapted heritage-based Serious Games. Cross-cultural studies could compare whether the same structural relationships hold across countries, whether measurement invariance can be established, and whether local cultural familiarity or cultural distance moderates the effects of heritage narrative involvement, perceived authenticity, and place attachment on anxiety reduction. Such work would clarify whether the cultural–psychological mechanism identified in this study represents a generalizable design principle or a mechanism whose strength and structure depend on specific cultural contexts.

### 7.5 Theoretical contributions and research limitations

The main theoretical contribution of this study is that it connects two lines of inquiry that have largely developed in parallel: Serious Games as psychological interventions and digital heritage as cultural experience. Whereas prior studies have generally framed games as intervention tools and cultural content as dissemination material, the present findings suggest that, in heritage-based Serious Games, cultural content may itself function as a psychological resource with emotion-regulatory value. By integrating the Stimulus–Organism–Response (SOR) framework and Self-Determination Theory (SDT), the study extends SOR from behavioral to emotional outcomes and clarifies the role of autonomy in cultural internalization, thereby shifting the analytical focus from cultural dissemination to cultural psychological function. Several limitations should nevertheless be acknowledged. First, the explanatory power for Flow and State Anxiety remained modest, suggesting that additional factors, including individual differences, situational stressors, and contextual conditions, are likely to be involved. Second, most core variables in this study were assessed using self-report measures. This approach was appropriate because the focal constructs, including Cultural Identity, Virtual Heritage Place Attachment, Flow, and State Anxiety, primarily refer to subjective psychological experiences that are difficult to capture fully through external observation alone. Nevertheless, reliance on self-report data may introduce common method bias, social desirability bias, and retrospective interpretation. Although the three-study design provided partial triangulation through experimental comparison, structural modeling, ANN-based prediction, and qualitative interviews, it cannot fully substitute for objective or longitudinal evidence. Future research should therefore incorporate behavioral indicators, gameplay log data, physiological measures, eye-tracking data, or longitudinal follow-up to further validate the proposed cultural–psychological mechanism. Third, because the study focused on a specific heritage-based Serious Game and a university-student sample, its generalizability to other cultural contexts, game types, and longer-term outcomes remains to be tested. Fourth, this study did not directly measure several alternative affective mechanisms that may contribute to anxiety reduction, such as nostalgia, emotional comfort, familiarity, escapism, and aesthetic pleasure. Future studies should include these variables to compare their relative explanatory power with the proposed cultural–psychological mechanism. Finally, because this study was conducted in a Chinese Intangible Cultural Heritage context, the generalizability of the findings to other cultural settings remains limited. The proposed mechanism may be transferable as a general cultural–psychological process, but its specific pathways and relative strengths may vary across countries, cultural traditions, and user groups. Future research should examine locally adapted heritage-based Serious Games in different cultural contexts and test whether the roles of Cultural Identity, Virtual Heritage Place Attachment, Flow, nostalgia, familiarity, and aesthetic pleasure differ across cultures. The present study should therefore be understood as identifying a core but bounded mechanism pathway that offers a theoretical basis and direction for future research.

## 8 Conclusion

This study moves beyond asking whether Serious Games reduce anxiety to examine how heritage-based Serious Games influence State Anxiety through culturally grounded psychological mechanisms. Across three studies integrating experimental, quantitative, and qualitative evidence, the findings show that heritage-based Serious Games can significantly reduce university students’ State Anxiety in the short term, with effects that exceed general game benefits. These emotional benefits appear to be partly achieved through internal psychological processes involving Cultural Identity, Virtual Heritage Place Attachment, and Flow, suggesting a structured pathway from cultural internalization to place-based meaning and immersive engagement. The study therefore reconceptualizes heritage-based Serious Games as culturally embedded experiential contexts with psychological consequences, rather than merely as tools of entertainment or dissemination. More broadly, it contributes to research on emotion regulation and digital interventions by demonstrating that cultural content can function as an active psychological resource. Nevertheless, the mechanism identified here should be regarded as core but bounded, and future research is needed to test its generalizability across different game types, cultural contexts, and longer-term outcomes.

## Data Availability

The raw data supporting the conclusions of this article will be made available by the authors, without undue reservation.
