# Supplementary material for: Beyond entertainment: cultural–psychological mechanisms underlying the reduction of state anxiety in heritage-based serious games
Source: Front Psychol. 2026 Aug 17;17:1854835. doi: 10.3389/fpsyg.2026.1854835 (PMC13526630; doi:10.3389/fpsyg.2026.1854835)
Supplement: Supplementary file 1 [file Data_Sheet_1.docx]

Supplementary Material

## 1. Supplementary Figures

## 1.1 Supplementary Figure 1

**Supplementary Figure 1.** Research model with explanatory sequential mixed-methods design.

## 1.2 Supplementary Figure 2

**Supplementary Figure 2.** Grounded Theory coding procedure

## 1.3 Supplementary Figure 3

**Supplementary Figure 3.** ANN models for A, B, C, and D

# 2 Supplementary Tables

## 2.1 Supplementary Table 1

**Supplemental Table 1.** Design-based matching strategy between the experimental and active-control conditions

| Dimension | Experimental condition | Active-control condition | control strategy |
| --- | --- | --- | --- |
| Game genre | Management-style Serious Game | Management-style Serious Game | Matched by game type |
| Core mechanics | Spatial construction, management, task progression | Spatial construction, management, task progression | Matched by gameplay structure |
| Gameplay duration | Approximately 30 minutes | Approximately 30 minutes | Controlled procedurally |
| Device format | Standardized mobile device | Standardized mobile device | Controlled procedurally |
| Experimental setting | Quiet experimental environment | Quiet experimental environment | Controlled procedurally |
| Task instruction | Neutral gameplay instruction | Neutral gameplay instruction | Controlled procedurally |
| Interaction process | Repeated interaction with game environment | Repeated interaction with game environment | Matched by interaction pattern |
| Operational load | Basic management and construction operations | Basic management and construction operations | Minimized through stimulus calibration |
| Interface clarity | Playable and understandable interface | Playable and understandable interface | Minimized through pilot calibration |
| General playability | Active gameplay condition | Active gameplay condition | Active-control design |
| Heritage narrative | Present | minimized | Intended manipulation |
| ICH-related content | Present | minimized | Intended manipulation |
| Heritage symbols | Present | minimized | Intended manipulation |
| Culturally meaningful spatial construction | Present | minimized | Intended manipulation |

## 2.2 Supplementary Table 2

## Supplementary Table 2. Demographic characteristics of respondents (N = 130)

| Variable | Category | Frequency | Percentage |
| --- | --- | --- | --- |
| Gender | Male | 58 | 44.62% |
|  | Female | 72 | 55.38% |
| Age | 21–22 | 32 | 24.62% |
|  | 23–24 | 61 | 46.92% |
|  | 25–26 | 27 | 20.77% |
|  | ≥27 | 10 | 7.69% |
| Education | Undergraduate | 78 | 60.00% |
|  | Master’s | 52 | 40.00% |
| Total |  | 130 | 100% |

## 2.3 Supplementary Table 3

**Supplementary Table 3.** Manipulation-check measures

Heritage Narrative Involvement

HNI1. During the gameplay, I felt drawn into the heritage-related storyline.

HNI2. The cultural cues in the game helped me enter the world it was trying to portray.

HNI3. The narrative progression made the heritage content feel meaningful to me.

Perceived Authenticity

PA1. The cultural content presented in the game felt authentic to me.

PA2. The scenes and symbols in the game seemed to be grounded in a credible heritage context.

PA3. The way the game presented cultural elements felt culturally convincing.

Perceived Autonomy

PAU1. I was able to make decisions in the game according to my own preferences.

PAU2. I felt free to arrange and manage the virtual space in my own way.

PAU3. My actions in the game felt self-directed rather than externally forced.

## 2.4 Supplementary Table 4

**Supplementary Table 4.** Participants’ demographic information (n=473).

| Attribute | Items | Numbers | Percentage (%) |
| --- | --- | --- | --- |
| Gender | Male | 268 | 52.86 |
|  | Female | 239 | 47.14 |
| Age | 18–22 | 195 | 38.46 |
|  | 23–26 | 168 | 33.14 |
|  | 27–29 | 97 | 19.13 |
|  | Other age groups | 47 | 9.27 |
| Education background | Bachelor's Degree | 208 | 41.03 |
|  | Master's Degree | 127 | 25.05 |
|  | Doctoral Degree | 52 | 10.26 |
|  | Staff | 120 | 23.67 |
| All | – | 507 | 100.00 |

## 2.5 Supplementary Table 5

**Supplementary Table 5.** Profile of interviewees.

| ID | Gender | Occupation | Primary Media Platforms Used |
| --- | --- | --- | --- |
| R1 | Female | Master’s student | Douyin |
| R2 | Male | Doctoral student | Xiaohongshu |
| R3 | Male | Internet industry professional | Douyin |
| R4 | Female | Doctoral student | Xiaohongshu |
| R5 | Female | Game developer | Douyin |
| R6 | Female | Master’s student | Xiaohongshu |
| R7 | Male | Internet industry professional | Douyin |
| R8 | Female | Freelancer | Douyin |
| R9 | Female | Content creator | Douyin |
| R10 | Male | Content creator | Xiaohongshu |
| R11 | Male | Internet industry professional | Xiaohongshu |
| R12 | Female | Master’ | Xiaohongshu |
| R13 | Female | Doctoral | WeChat |
| R14 | Female | Game developer | Xiaohongshu |
| R15 | Male | Media and cultural industry professional | WeChat |
| R16 | Female | Internet industry professional | Weibo |
| R17 | Male | Doctoral student | Xiaohongshu |
| R18 | Male | Professional gamer | Gaming websites |
| R19 | Female | Freelancer | Douyin |
| R20 | Female | Master’s student | Douyin |
| R21 | Male | Professional gamer | Gaming communities |
| R22 | Male | Internet industry professional | Xiaohongshu |
| R23 | Male | Freelancer | Douyin |
| R24 | Male | Master’s student | Gaming websites |
| R25 | Female | Media and cultural industry professional | Xiaohongshu |
| R26 | Female | Game developer | Gaming communities |
| R27 | Female | Game developer | Gaming communities |
| R28 | Female | Content creator | WeChat |

## 2.6 Supplementary Table 6

**Supplemental Table 6. Semi-structured interview questions.**

Pre-question

R1. * Are you currently able to clearly recall your gameplay experience with this game, as well as your primary feelings when completing the questionnaire? *

Demographic questions

Q1: Could you briefly describe your occupation and generational cohort (Gen Y or Gen Z)?

Q2: Which social media platform do you use most frequently in your daily life (Xiaohongshu, Douyin, gaming communities, gaming websites, WeChat)?

Heritage Narrative Involvement

Q3: To what extent did the game’s narrative context, cultural cues, or storyline progression draw you into the game world? Could you provide specific examples or moments that illustrate this experience?

Q4: Do you perceive these narrative elements as integral components of the overall experience, or more as background embellishments? Why?

Perceived Authenticity

Q5: How do you perceive the cultural content, spatial settings, and cultural symbols presented in the game? Do they feel authentic to you, or do they appear to be culturally grounded?

Q6: During your gameplay experience, were there specific moments that made the game feel particularly “credible” or “culturally authentic,” or conversely, moments when it felt less convincing? What factors influenced your judgment?

Perceived Autonomy

Q7: During the process of constructing, arranging, or managing the virtual space, did you feel that you were able to make decisions according to your own preferences?

Q8: Do you feel that the game supports your autonomy in making choices and exploring, or does it primarily guide you along predetermined paths? How did this perception influence your overall experience?

Cultural Identity

Q9: During your gameplay experience, did the cultural content at any point begin to feel personally relevant or meaningful to you?

Q10: Did you experience a transition from merely noticing cultural elements to genuinely identifying with or valuing them? If so, how did this change occur?

Virtual Heritage Place Attachment

Q11: As your experience progressed, did you develop any sense of attachment, belonging, or emotional connection to the virtual heritage space? Why?

Q12: For you, did this virtual space remain merely a functional game environment, or did it gradually become a “meaningful place”? What factors contributed to this difference?

Flow

Q13: During gameplay, did you experience moments of intense focus, a loss of time awareness, or deep immersion?

Q14: What do you think primarily contributed to this sense of immersion—the tasks themselves, the process of spatial construction, the cultural content, or a combination of these factors?

State Anxiety

Q15: After completing the gameplay experience, did you notice any changes in your levels of tension, worry, or stress? If so, how would you describe these changes?

Q16: Do you consider these emotional changes to be primarily a form of short-term relaxation, or are they more closely related to your experiences with cultural content, spatial engagement, or immersion? Why?

Final Reflections

Q17. Compared with ordinary games, in what ways do you think heritage-based Serious Games are more likely to influence players’ emotional states? Are there any other important aspects that we have not yet discussed?

Notes: * = All participants were able to recall their responses from Study 1.

Source: Authors’ own compilation.
